# Cancer Cell Cytotoxicities of 1-(4-Substitutedbenzoyl)-4-(4-chlorobenzhydryl)piperazine Derivatives

**DOI:** 10.3390/ijms13078071

**Published:** 2012-06-28

**Authors:** Mine Yarim, Meric Koksal, Irem Durmaz, Rengul Atalay

**Affiliations:** 1 Department of Pharmaceutical Chemistry, Faculty of Pharmacy, Yeditepe University, 34755, Kayisdagi, Istanbul, Turkey; E-Mail: merickoksal@yeditepe.edu.tr; 2 Department of Molecular Biology and Genetics, BilGen, Genetics and Biotechnology Research Center, Faculty of Science, Bilkent University, 06800, Bilkent, Ankara, Turkey; E-Mails: irem.durmaz@bilkent.edu.tr (I.D.); rengul@bilkent.edu.tr (R.A.)

**Keywords:** 1-(4-chlorobenzhydryl)piperazine derivatives, cell proliferation, benzoyl chlorides, cytotoxicity, cancer

## Abstract

A series of novel 1-(4-substitutedbenzoyl)-4-(4-chlorobenzhydryl)piperazine derivatives **5a**–**g** was designed by a nucleophilic substitution reaction of 1-(4-chlorobenzhydryl)piperazine with various benzoyl chlorides and characterized by elemental analyses, IR and ^1^H nuclear magnetic resonance spectra. Cytotoxicity of the compounds was demonstrated on cancer cell lines from liver (HUH7, FOCUS, MAHLAVU, HEPG2, HEP3B), breast (MCF7, BT20, T47D, CAMA-1), colon (HCT-116), gastric (KATO-3) and endometrial (MFE-296) cancer cell lines. Time-dependent cytotoxicity analysis of compound **5a** indicated the long-term *in situ* stability of this compound. All compounds showed significant cell growth inhibitory activity on the selected cancer cell lines.

## 1. Introduction

Cancer still continues to be the leading cause of deaths worldwide and thus there is a pressing need for novel and effective treatments. Cancer is the disease resulting from abnormal cells with abilities of uncontrolled dividing and invasion to other tissues through blood and lymph systems. Despite major breakthroughs in many areas of modern medicine over the past 100 years, the successful treatment of cancer remains a significant challenge at the start of the 21st century. Because of its general toxicity, chemotherapy has a limited use in cancer treatment. Novel agents that selectively kill tumor cells or inhibit their proliferation without being generally toxic have yet to be discovered. In the field of chemotherapeutic drugs, the search for new, more active, more selective and less toxic compounds is still very intense, and new, promising anticancer approaches are being tested [1,2]. Currently, combined anticancer therapies or multi-acting drugs are clinically preferred to traditional cytotoxic treatment, with the aim of avoiding resistance and toxicity drawbacks. These issues often prevent successful treatment and are responsible for reduced survival times [3,4]. Mass screening of synthetic derivatives and natural products over the last 50 years has led to the discovery of the currently utilized anticancer drugs.

Piperazines are currently the most important building blocks in drug discovery, with a high number of positive hits encountered in biological screens of this heterocycle and its congeners. The piperazine template forms the molecular backbone, possesses versatile binding properties with a frequently occurring binding motif, and provides potent and selective ligands for a range of different biological targets in medicinal chemistry. The piperazine scaffold and its analogues are important pharmacophores that can be found in biologically active compounds across a number of different therapeutic areas [5,6]. These include anticancer [7–9], antifungal [10], antibacterial, antimalarial and antipsychotic agents [11], as well as HIV protease inhibitors [12–14] and antidepressants [15]. MST-16 [4,4-1,2-(ethanediyl)bis(1-isobutoxycarbonyloxy-methyl-2,6-piperazinedione)] was recently approved as an oral anticancer drug for clinical use in Japan [16]. The piperazine analogues have shown potent antiproliferative activity against colon, prostate, breast, lung and leukemia tumors; additional studies by the U.S. National Cancer Institute (NCI) have demonstrated the ability of the lead piperazines to suppress and eliminate experimental tumors in small-animal models. Mechanistic evaluations have shown that piperazines inhibit microtubule synthesis, inhibit cell cycle progression and inhibit angiogenesis, which is critical to a tumor cell’s ability to grow and metastasize. Piperazines kill tumor cells directly through the induction of apoptosis. Their anti-tumor mode of action is quite distinct from that of Taxol and, compared to that drug, they are significantly more potent, are active against a variety of different tumor types, and are orally bioavailable. In the literature, we found that diaryl piperazine derivatives were identified as potent and selective dopamine D4 receptor antagonists [17–19], enterovirus inhibitors [20] and inhibitors of dopamine uptake in the central nervous system [6,21–23]. Piperazine sulfonamides exhibit diverse pharmacological activity such as MMP-3 enzyme inhibition and carbonic anhydrase inhibition [20,24]. Piperazine derivatives were shown to inhibit growth inhibition of human erythroleukemia K562 cells and myeloid leukemia HL-60 cells [7] and also shown to inhibit topoisomarase II activity [25]. Wilson *et al.* reported the interaction of DNA with an unfused aromatic system containing terminal piperazino substituents [26]. Sampson J.J. *et al.* reported a piperazine derivative induces apoptosis in U937 cells [27]. *N*-Alkyl, *N*-sulfonyl and *N*-benzoyl derivatives of benzhydrylpiperazine derivatives show antimicrobial and anticancer activity [28–30].

We describe here the synthesis of 1-(4-substitutedbenzoyl)-4-(4-chloro-benzhydryl)piperazine derivatives and their effect on the inhibition of cancer cell lines from liver (HUH7, FOCUS, MAHLAVU, HEPG2, HEP3B), breast (MCF7, BT20, T47D, CAMA-1), colon (HCT-116), gastric (KATO-3) and endometrial (MFE-296) samples.

## 2. Results and Discussion

### 2.1. Chemistry

**Scheme 1:**
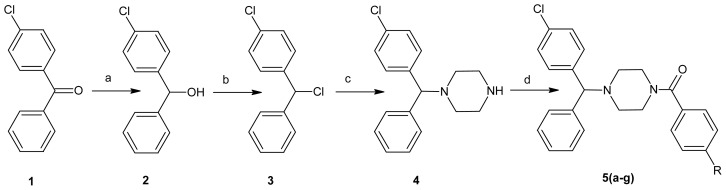
Reagent and conditions: (**a**) NaBH_4_, methanol-THF, room temperature, 2 h; (**b**) thionyl chloride, methylene dichloride (MDC), room temperature, overnight; (**c**) piperazine, MeCN, 90 °C, 16 h; (**d**) R-Ph-COCl, MDC, triethylamine, room temperature, 6–7 h.

1-(4-Chlorobenzhydryl)piperazine derivatives **5a**–**g** were prepared by the method summarized in Scheme 1. First, compound **2**, benzhydrol, was synthesized by reduction of benzophenone **1** using sodium borohydride and achieved a 92% yield. Compound **2** was subsequently treated with thionyl chloride to give the corresponding 4-chlorobenzhydryl chloride **3**, which was directly reacted with piperazine and anhydrous potassium carbonate using dimethyl formamide as a solvent at 80 °C to give the target key intermediate 1-(4-chlorobenzhydryl)piperazine **4**. The nucleophilic substitution reactions of **4** with different benzoyl chlorides (Ph-CO-Cl) were carried out in the presence of triethylamine and methylene dichloride (MDC) as the solvent, with a good yield (73%–90%) and good purity. The crude products were purified by column chromatography over silica gel using hexane:ethyl acetate (8:2) as an eluent. Hydrogen chloride gas was introduced into the solution and the hydrochloride salt of final compounds **5a**–**g** precipitated from the organic phase.

Synthesized molecules **5a**–**g** were structurally characterized by elemental analyses, IR and nuclear magnetic resonance spectra. The chemical structures and physical data of all the synthesized compounds are given in Table 1.

The compounds were prepared by the reactions of 1-(4-chlorobenzhydryl)piperazine with different acid chlorides containing substituted aromatic rings. The N-substitution of 1-(4-chlorobenzhydryl)piperazine with different benzoyl chlorides was confirmed by the disappearance of the N-H group in IR and ^1^H NMR data. The stretching frequency of N–C=O at 1635–1645 cm^−1^ on IR spectra was also supported the formation of compounds **5a**–**g**. The obtained products were purified by column chromatography using hexane:ethyl acetate (8:2) as an eluent. Hydrogen chloride gas was introduced into the solution and the hydrochloride salt of final compounds **5a**–**g** precipitated from the organic phase. The new derivatives of 1-(4-chlorobenzhydryl)piperazine benzamide **5a**–**g** were tested for their effect on cellular viability against cancer cell lines from liver (HUH7, FOCUS, MAHLAVU, HEPG2, HEP3B), breast (MCF7, BT20, T47D, CAMA-1), colon (HCT-116), gastric (KATO-3) and endometrium (MFE-296) samples. The results are given in Table 2.

### 2.2. Cytotoxicity Analysis of the Compounds

**Figure 1:**
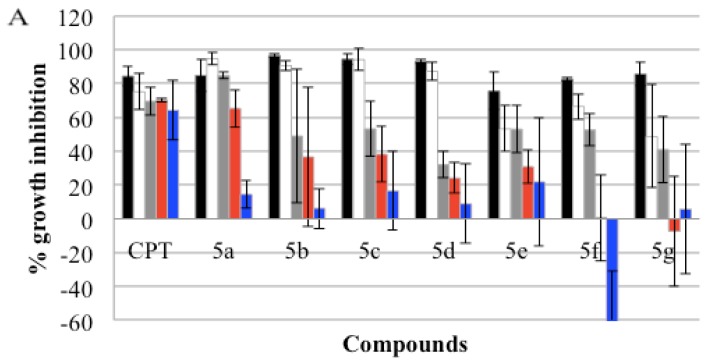

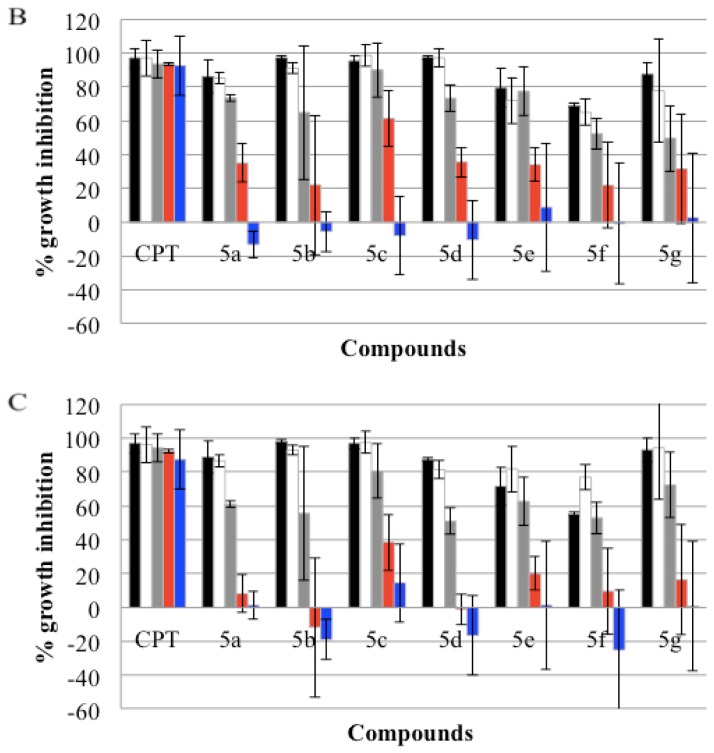
Percent growth inhibition graphs of compounds **5a**–**g** on HUH7 (**A**), HCT116 (**B**) and MCF7 (**C**) cell lines. The cytotoxicity was investigated using the SRB assay after the cells were treated with the corresponding compounds and incubated for 72 h at 40 μM (black), 20 μM (white), 10 μM (grey), 5 μM (red) and 2.5 μM (blue) concentrations. All the experiments were conducted in triplicate. DNA topoisomerase I inhibitor Camptothecin (CPT) was applied with the same serial dilutions as the compounds, from 40 μM to 2.5 μM (black, white, grey, red, and blue), as a positive control.

The cytotoxic activity of the synthesized compounds was investigated initially on liver (HUH7), breast (MCF7) and colon (HCT116) cancer cell lines, by means of sulphorhodamine B (SRB) assays [31] in triplicate. Serial dilutions from 40 μM to 2.5 μM were used and Camptothecin was the positive control for the cytotoxic effect (Table 2, Figure 1). As seen in Table 2, all compounds showed high cytotoxicity levels on the selected cancer cell lines. A 50% growth inhibition of the cancer cell lines was observed in micromolar concentrations. According to these promising results, the carcinoma cell lines for the compounds were enlarged in liver (FOCUS, MAHLAVU, HEPG2, HEP3B), breast (BT20, T47D, CAMA-1), gastric (KATO-3) and endometrial (MFE-296) (Table 2) samples. The compounds also showed an inhibitory effect on additional cell lines. **5a**, **5b** and **5c** especially had lower GI_50_ values when compared to 5-fluorouracil (5-FU).

Among compounds **5a**–**g**, the best inhibitory activity against HUH7 (GI_50_ = 4.64) and FOCUS (GI_50_ = 4.15) was exhibited by compound **5a**, which had a para chloro substitution on benzoyl moiety (Table 1). For HEPG2 (GI_50_ = 7.22), HEP3B (GI_50_ = 1.67), MCF7 (GI_50_ = 6.09), BT20 (GI_50_ = 11.62), CAMA-1 (GI_50_ = 1.22), HCT116 (GI_50_ = 6.18), KATO-3 (GI_50_ = 10.07), MFE-296 (GI_50_ = 9.73) cell lines, compound **5c**, containing the para methoxy group showed the lowest GI_50_ values. For the remaining cell lines T47D (GI_50_ = 0.31) and MAHLAVU (GI_50_ = 7.00), compounds **5e** (R: NO_2_) and **5g** (R: 2,4-di F) represented the best results, respectively. It has been observed that all the compounds had lower GI_50_ values than the reference drug 5-FU against HUH7, HEP3B, T47D and HCT116. **5a**, **5c**, **5e**, **5g** against T47D, and **5c** against HEP3B and CAMA-1 especially showed significantly important low GI_50_ values: 1.91, 0.44, 0.31, 0.85, 1.67 and 1.22, respectively.

Compound **5c** had better results in most of the carcinoma cell lines than the others in this series. By structural assessment, introducing electron-donating methoxy groups to the phenyl ring of the substituent at the para position (**5c**) resulted in an increase in activity against all cell lines. Compounds having halogen substituents (**5a**, **5b** and **5d**) also represented promising GI_50_ values against most of the cell lines. Introduction of a second fluorine atom to the benzoyl moiety (**5g**) changed GI_50_ values. Although there were no correlations between the values, the GI_50_ values were significantly decreased for the FOCUS, MAHLAVU, T47D, CAMA-1 and HCT-116 cell lines. Compound **5f**, containing biphenyl moiety on a piperazine ring, showed less inhibitory activity against most of the carcinoma cell lines. This lower inhibition might be due to the presence of a bulky group like biphenyl.

#### Real Time Cytotoxicity Analysis of Compound **5a**

**Figure 2:**
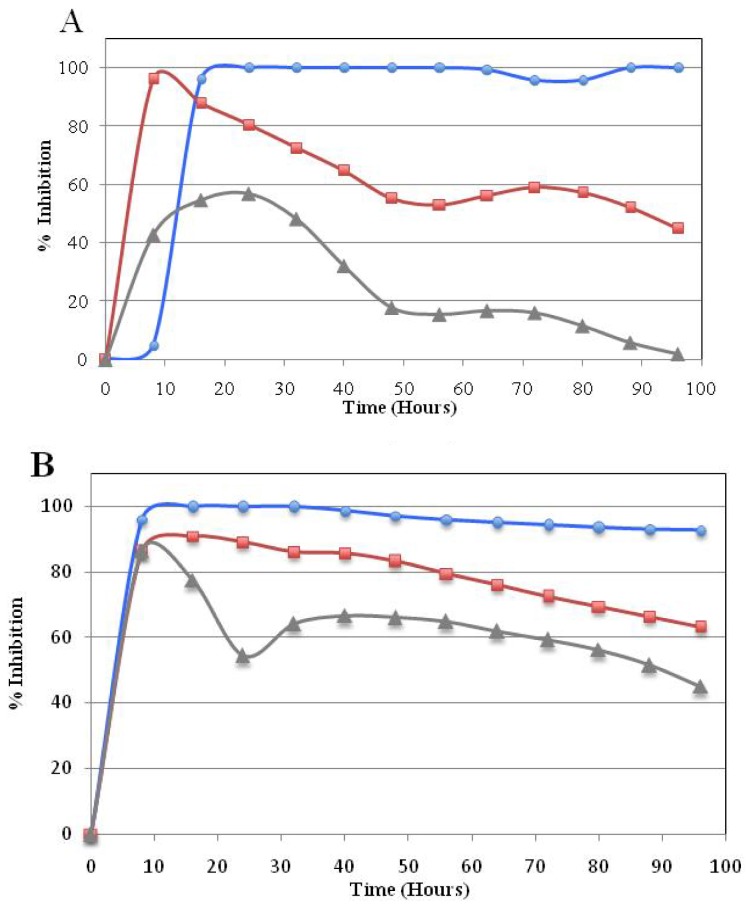

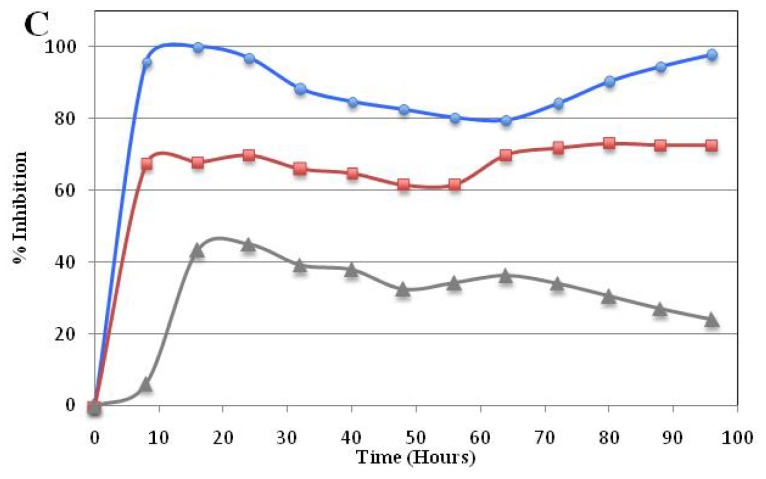
Real-time cell growth surveillance in the presence of compound **5a**. The compound 5**a** was applied on HUH7 (**A**), HCT116 (**B**) and MCF7 (**C**) cell lines in GI_100_( 
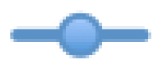
), GI_50_( 
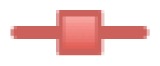
), and GI_25_ ( 
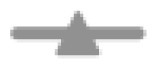
) concentrations, which were calculated from Table 2. Real-time cell growth surveillance by the cell impedance-based xCELLigence data acquisition system was performed in triplicate.

In this continuation study, the long term cytotoxic effects of promising compound **5a** on the cancer cell lines were analyzed in real-time cell growth surveillance by a cell electronic sensing assay (xCelligence). The growth inhibitory effect of compound **5a** on the HUH7, HCT-116 and MCF-7 cell lines was dynamically monitorized for 96 h. The compound was tested in triplicates of three concentrations (GI_100_, GI_50_ and GI_25_) that were obtained from the SRB assay for each specific cell line. The data was collected every 30 min. The cell growth in treated wells was normalized to DMSO-containing wells and a growth inhibition curve was created. The curve showed a time-dependent effect of the compound in different concentrations in all three cell lines (Figure 2). The cell growth inhibition curves demonstrated that after 24 h of the cytotoxic activity, compound **5a** had an irreversible growth-inhibitory effect parallel with the inhibitory concentrations given. Compound **5a** had a permanent irreversible effect especially on the liver cancer cell line HUH7 with GI_100_ concentrations (Figure 2A).

## 3. Experimental Section

### 3.1. Chemistry

Melting points (°C) were determined by using a Mettler-Toledo FP62 capillary melting point apparatus (Mettler-Toledo, Greifensee, Switzerland) and are uncorrected. Infrared spectra were recorded on a Perkin-Elmer Spectrum One series FTIR apparatus (Version 5.0.1) (Perkin Elmer, Norwalk, CT, USA), using potassium bromide pellets. The frequencies are expressed in cm^−1^. The ^1^H-NMR spectra were recorded with a Varian Mercury-400 FT-NMR spectrometer (Varian Inc., Palo Alto, CA, USA), using tetramethylsilane as the internal reference, with chloroform-CDCl_3_ or dimethylsulphoxide-DMSO-d6 as solvents. The chemical shifts are reported in parts per million (ppm). Elemental analyses were performed on a LECO 932 CHNS (Leco-932, St. Joseph, MI, USA) instrument and were within ± 0.4% of the theoretical values.

#### 3.1.1. General Procedure for the Synthesis of 1-(4-Chlorobenzhydryl)piperazine (**4**)

4-Chlorobenzophenone (50 mmol) was dissolved in a mixture of methanol (100 mL) and THF (150 mL) and cooled to 0 °C. NaBH_4_ (50 mmol) was added to the above solution at 0 °C. After an additional 10 min at 0 °C, the reaction mixture was subsequently stirred at room temperature for 2 h. The reaction mixture was diluted with water (200 mL), and the product was extracted with diethylether (400 mL). The organic phase was washed with 1 N HCl, followed by a saturated NaHCO_3_ and finally with water. It was dried over MgSO_4_ and evaporated under vacuum to provide 4-chlorobenzhydrol (compound **2**, Yield: 92%). The crude product was used in the following steps without further purification.

The alcohol (compound **2**, 20 mmol) was dissolved in MDC (50 mL) and SOCl_2_ (22 mmol) was added to the solution. The reaction mixture was stirred at room temperature overnight and the solvent was evaporated under vacuum (compound **3**). The crude residue was dissolved in MeCN (100 mL) and piperazine (200 mmol) was added. The mixture was refluxed at 90 °C for 16 h and monitored by TLC. The solvent was evaporated under vacuum and the residue was taken in water and extracted with ethyl acetate. Finally a water wash was given to the organic layer, followed by drying with anhydrous sodium sulphate. The solvent was evaporated to obtain the crude product, which was purified by column chromatography over silica gel (60–120 mesh) using chloroform:methanol (9:1) as the eluent (compound **4**, yield: 70%).

#### 3.1.2. General Procedure for the Synthesis of 1-(4-Substitutedbenzoyl)-4-(4-chlorobenzhydryl)piperazine Hydrochloride Salts **5**(**a**–**g**)

A solution of 1-(4-chlorobenzhydryl)piperazine 6 (1.98 mmol) in dry dichloromethane was taken and cooled to 0–5 °C in an ice bath. Triethylamine (5.94 mmol) was added to the cold reaction mixture and stirred for 10 min, and then different benzoyl chlorides (1.98 mmol) were added. The reaction mixture was stirred for 5–6 h at room temperature, and monitored by TLC. Upon completion, the solvent was removed under reduced pressure and residue was taken in water and extracted with ethyl acetate. The organic layer was washed with 10% ammonium chloride solution and finally a water wash was given to the organic layer and dried with anhydrous sodium sulphate. The solvent was evaporated to obtain the crude product, which was purified by column chromatography over silica gel (60–120 mesh) using hexane:ethyl acetate (8:2) as the eluent. Hydrogen chloride gas was introduced into the solution and the hydrochloride salt of the final compounds (**5a**–**g**) precipitated from the ether.

##### 3.1.2.1. 1-(4-Chlorobenzoyl)-4-(4-chlorobenzhydryl)piperazine Hydrochloride Salt (**5a**)

The general synthetic method described above afforded **5a**, and the product obtained was in white crystalline solid form from 1-(4-chlorobenzhydryl)piperazine (1.98 mmol) and 4-chlorobenzoyl chloride (1.98 mmol). IR (KBr, cm^−1^): 3029, 2961, 2889, 1636, 1350. ^1^H-NMR (DMSO, 400 MHz) 1.96 (br s, 2H, –CH_2_–), 2.86 (br s, 2H, –CH_2_–), 3.47 (br s, 2H, –CH_2_–), 3.63 (br s, 2H, –CH_2_–), 4.93 (s, 1H, –CH), 7.37–7.45 (m, 8H, 2 4-ClPh), 7.92 (s, 5H, Ph), 13.66 (s, 1H, NH). MS (ESI, + ion): *m*/*z* = 460.09 Anal. calcd. for C_24_H_23_Cl_3_N_2_O (in %): C 62.42, H 5.02, N 6.07. Found C 62.38, H 5.01, N 6.00.

##### 3.1.2.2. 1-(4-Fluorobenzoyl)-4-(4-chlorobenzhydryl)piperazine Hydrochloride Salt (**5b**)

The general synthetic method described above afforded **5b**, and the product obtained was in white crystalline solid form from 1-(4-chlorobenzhydryl)piperazine (1.98 mmol) and 4-fluorobenzoyl chloride (1.98 mmol). IR (KBr, cm^−1^): 3009, 2892, 1636, 1033. ^1^H-NMR (DMSO, 400 MHz) 2.26 (br s, 2H, –CH_2_–), 2.90 (br s, 2H, –CH_2_–), 3.48 (br s, 2H, –CH_2_–), 4.24 (br s, 2H, –CH_2_–), 4.99 (s, 1H, –CH), 7.06–7.10 (m, 4H, 4-ClPh), 7.35–7.45 (m, 4H, 4-FPh), 7.93 (s, 5H, Ph), 13.57 (s, 1H, NH). MS (ESI, + ion): *m*/*z* = 444.12 Anal. calcd. for C_24_H_23_Cl_2_FN_2_O (in %): C 64.72, H 5.21, N 6.29. Found C 64.68, H 5.11, N 6.25.

##### 3.1.2.3. 1-(4-Methoxybenzoyl)-4-(4-chlorobenzhydryl)piperazine Hydrochloride Salt (**5c**)

The general synthetic method described above afforded **5c**, and the product obtained was in white crystalline solid form from 1-(4-chlorobenzhydryl)piperazine (1.98 mmol) and 4-methoxybenzoyl chloride (1.98 mmol). IR (KBr, cm^−1^): 3029, 2891, 1636, 1237, 1112. ^1^H-NMR (DMSO, 400 MHz) 2.41 (br s, 2H, –CH_2_–), 2.86 (br s, 2H, –CH_2_–), 3.44 (br s, 2H, –CH_2_–), 3.81 (s, 3H, –OCH_3_), 4.24 (br s, 2H, –CH_2_–), 4.93 (s, 1H, –CH), 6.87–76.91 (m, 4H, 4-ClPh), 7.34–7.44 (m, 4H, 4-OCH_3_Ph), 7.92 (s, 5H, Ph), 13.56 (s, 1H, NH). MS (ESI, + ion): *m*/*z* = 456.14 Anal. calcd. for C_25_H_26_Cl_2_N_2_O (in %): C 65.65, H 5.73, N 6.12. Found C 65.48, H 5.70, N 6.11.

##### 3.1.2.4. 1-(4-Bromobenzoyl)-4-(4-chlorobenzhydryl)piperazine Hydrochloride Salt (**5d**)

The general synthetic method described above afforded **5d**, and the product obtained was in white crystalline solid form from 1-(4-chlorobenzhydryl)piperazine (1.98 mmol) and 4-bromobenzoyl chloride (1.98 mmol). IR (KBr, cm^−1^): 3029, 2935, 1636, 688. ^1^H-NMR (DMSO, 400 MHz) 2.90 (br s, 2H, –CH_2_–), 3.47 (br s, 2H, –CH_2_–), 4.01 (br s, 2H, –CH_2_–), 4.31 (br s, 2H, –CH_2_–), 5.10 (s, 1H, –CH), 7.28–7.30 (m, 4H, 4-BrPh), 7.37–7.42 (m, 4H, 4-FPh), 7.94 (s, 5H, Ph), 13.44 (s, 1H, NH). ^13^C-NMR (400 MHz, CDCl_3_, ppm): 169.60, 136.13, 133.76, 132.79, 132.63, 132.28, 130.38, 130.26, 130.20, 130.11, 129.13, 128.76, 125.42, 77.74, 77.64, 77.32, 76.99, 52.35 MS (ESI, + ion): *m*/*z* = 504.04 Anal. calcd. for C_24_H_23_BrCl_2_N_2_O (in %): C 56.94, H 4.58, N 5.53. Found C 56.82, H 4.55, N 5.51.

##### 3.1.2.5. 1-(4-Nitrobenzoyl)-4-(4-chlorobenzhydryl)piperazine Hydrochloride Salt (**5e**)

The general synthetic method described above afforded **5e**, and the product obtained was in white crystalline solid form from 1-(4-chlorobenzhydryl)piperazine (1.98 mmol) and 4-nitrobenzoyl chloride (1.98 mmol). IR (KBr, cm^−1^): 3073, 2961, 1643, 1358, 1283. ^1^H-NMR (DMSO, 400 MHz) 2.68 (br s, 2H, –CH_2_–), 3.00 (br s, 2H, –CH_2_–), 3.50 (br s, 2H, –CH_2_–), 4.11 (br s, 2H, –CH_2_–), 5.13 (s, 1H, –CH), 7.39–7.44 (m, 4H, 4-ClPh), 7.62–7.64 (m, 4H, 4-NO_2_Ph), 7.94 (s, 5H, Ph), 13.36 (s, 1H, NH). ^13^C-NMR (400 MHz, CDCl_3_, ppm): 168.25, 149.14, 140.05, 136.36, 133.49, 132.33, 130.30, 128.70, 128.60, 124.37, 52.35 MS (ESI, + ion): *m*/*z* = 471.11 Anal. calcd. for C_24_H_23_Cl_2_N_3_O_3_ (in %): C 61.02, H 4.91, N 8.90. Found C 61.00, H 4.88, N 8.89.

##### 3.1.2.6. 1-(4-Phenylbenzoyl)-4-(4-chlorobenzhydryl)piperazine Hydrochloride Salt (**5f**)

The general synthetic method described above afforded **5f**, and the product obtained was in white crystalline solid form from 1-(4-chlorobenzhydryl)piperazine (1.98 mmol) and biphenyl-4-carbonyl chloride (1.98 mmol). IR (KBr, cm^−1^): 3053, 2941, 1635, 1327, 1280. ^1^H-NMR (DMSO, 400 MHz) 2.54 (br s, 2H, –CH_2_–), 2.90 (br s, 2H, –CH_2_–), 3.48 (br s, 2H, –CH_2_–), 4.24 (br s, 2H, –CH_2_–), 4.97 (s, 1H, –CH), 7.06–7.10 (m, 4H, 4-ClPh), 7.37–7.60 (m, 9H, biphenyl), 7.93 (s, 5H, Ph), 13.58 (s, 1H, NH). MS (ESI, + ion): *m*/*z* = 502.16 Anal. calcd. for C_30_H_28_Cl_2_N_2_O (in %): C 71.57, H 5.61, N 5.56. Found C 71.53, H 5.59, N 5.51.

##### 3.1.2.7. 1-(2,4-Difluorobenzoyl)-4-(4-chlorobenzhydryl)piperazine Hydrochloride Salt (**5g**)

The general synthetic method described above afforded **5g**, and the product obtained was in white crystalline solid form from 1-(4-chlorobenzhydryl)piperazine (1.98 mmol) and 2,4-difluorobenzoyl chloride (1.98 mmol). IR (KBr, cm^−1^): 2923, 2852, 1644, 1167. ^1^H-NMR (DMSO, 400 MHz) 3.02 (br s, 2H, –CH_2_–), 3.49 (br s, 2H, –CH_2_–), 4.05 (br s, 2H, –CH_2_–), 4.69 (br s, 2H, –CH_2_–), 5.27 (s, 1H, –CH), 6.79–6.97 (m, 4H, 4-ClPh), 7.31–7.44 (m, 3H, 2,4-diFPh), 7.99 (s, 5H, Ph), 13.38 (s, 1H, NH). ^13^C-NMR (400 MHz, CDCl_3_, ppm): 165.50, 164.40, 163.01, 159.90, 157.40, 136.01, 133.91, 132.79, 131.30, 130.16, 128.78, 118.80, 113.70, 104.52, 77.50, 52.50, 43.48, 38.50, 31.76, 22.83, 15.47, 14.33 MS (ESI, + ion): *m*/*z* = 462.11 Anal. calcd. for C_24_H_22_Cl_2_F_2_N_2_O (in %): C 62.21, H 4.79, N 6.05. Found C 62.13, H 4.75, N 6.02.

### 3.2. Biology

#### 3.2.1. Cell Culture

The human cancer cell lines (except KATO-3 and MFE-296) were grown in Dulbecco’s Modified Eagle’s Medium (DMEM), with 10% fetal bovine serum (FBS) and 1% penicillin and incubated in 37 °C incubators containing 5% CO_2_ and 95% air. The KATO-3 gastric cancer cell lines were grown in high-glucose DMEM (4.5g/L glucose) with 10% FBS, 1% penicillin, 1% l-glutamine and 1% non-essential amino acid. The MFE-296 endometrial cancer cell lines were grown in a medium containing 40% RPMI 1640, 40% minimum essential medium (MEM) (with Earle’salts), 20% FBS, 2mM l-glutamine and 1× insulin-transferrin-sodium selenite.

#### 3.2.2. NCI-60 Sulphorhodamine B Assay

Cancer cells (range of 2000cell/well to 5000cell/well) were inoculated into 96-well plates in 200 μL of media and incubated in 37 °C incubators containing 5% CO_2_ and 95% air. After a 24 h incubation period, one plate for each cell line was fixed with 100 μL 10% ice-cold trichloroacetic acid (TCA). This plate represents the behavior of the cells just prior to drug treatment and is accepted as the time-zero plate. The compounds to be tested were solubilized in dimethyl sulfoxide (DMSO) to a final concentration of 40 mM and stored at 4 °C. While treating the cells with the compounds, the corresponding volume of the compound was applied to the cell to achieve the desired drug concentration and diluted through serial dilution. After drug treatment, the cells were incubated in 37 °C incubators containing 5% CO_2_ and 95% air for 72 h. Following the termination of the incubation period after drug treatment, the cells were fixed with 100 μL 10% ice-cold TCA and incubated in the dark at 4 °C for 1 h. Then the TCA was washed away with dH_2_O five times and the plates were left to air dry. For the final step, the plates were stained with 100 μL of 0.4% SRB (cat.86183—5 g from Sigma) solution in 1% acetic acid solution. Following staining, the plates were incubated in dark for 10 min at room temperature. The unbound dye was washed away using 1% acetic acid and the plates were left to air dry. To measure the absorbance results, the bound stain was then solubilized using 200 μL of 10 mM Tris-Base. The OD values were obtained at 515 nm.

#### 3.2.3. Time-dependent Cellular Response Profiles by Cell Electronic Sensing (xCELLigence)

Media (50 μL) was applied to each well of the E-plate and a background absorbance was measured in order to eliminate any background noise. Then, the HUH7, HCT-116 and MCF7 cell lines (5000 cell/well in 150 μL) were inoculated into E-plates (96 well) (Roche). The proliferation curve of the cells were observed in real-time cell electronic sensing RT-CES (xCELLigence-Roche Applied Science). For the first 24-h period, the cell index was measured every 30 min [32]. After a 24 h incubation, the 150 μL medium was replaced with 100 μL fresh medium in each well. Compound **5a** was then applied to each cell with the indicated concentrations. For the first 24 h, the short-term drug response was monitored by taking the cell index values every 10 min. Then, to monitor the long-term drug response, cell index values were taken every 30 min. Cell index values represent the impedance measurements and these values are used in calculating the inhibitory effect of the compound by calculating CI_drug_/CI_DMSO_ [33].

## 4. Conclusion

Currently, a large variety of chemotherapeutic drugs are used to treat cancer. However, many compounds have limited efficacy due to problems of delivery and penetration and a moderate degree of selectivity for cancer cells. In this study, our results demonstrate that the synthesized compounds **5a**–**g** exhibit a high cytotoxic effect on growing cancer cells *in vitro*. In addition, a time-dependent cytotoxicity analysis of compound **5a** demonstrates that after penetrating into the cell this compound has a long-term effect, which is an indication of stable *in situ* activity. This study identifies this new series of agents for cancer therapy. All the compounds tested in this study are racemates, suggesting that further gains in potency may be realized by resolving the enantiomers. Enantioselectivity is a key measure of specificity in evaluating mechanisms of action and would provide much-needed understanding of the function of these compounds in inhibiting cancer cell growth. These results represent a promising start point for our continuous studies.

## Figures and Tables

**Table 1:** Chemical structures, physical data of synthesized compounds.

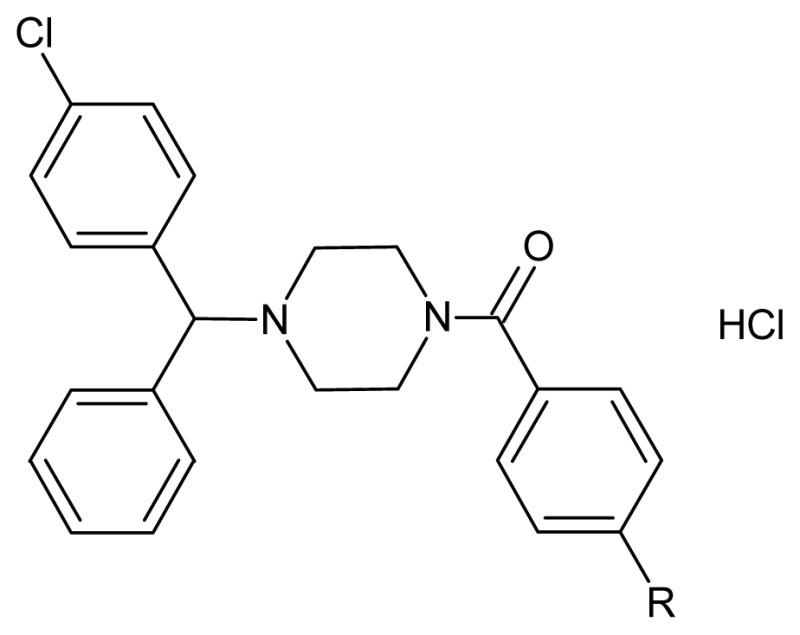				
Compounds	R	Formula	Yield (%)	m.p. (°C)
5a a	Cl	C_24_H_23_Cl_3_N_2_O	75	172.8
5b a	F	C_24_H_23_Cl_2_FN_2_O	81	144. 2
5c	OCH_3_	C_25_H_26_Cl_2_N_2_O_2_	73	132.3
5d	Br	C_24_H_23_BrCl_2_N_2_O	75	155.3
5e	NO_2_	C_24_H_23_Cl_2_N_3_O_3_	76	153.4
5f	Ph	C_30_H_28_Cl_2_N_2_O	70	152.1
5g a	2,4-di F	C_24_H_22_Cl_2_F_2_N_2_O	90	215.5 decomp

a CAS registry numbers for **5a**: 924504-29-0, **5b**: 924494-00-8, **5g**: 924519-13-1.

**Table 2:** GI_50_ (μM) of 5a-g for liver (HUH7, FOCUS, MAHLAVU, HEPG2, and HEP3B), breast (MCF7, BT20, T47D, and CAMA-1), colon (HCT116), gastric (KATO-3) and endometrial (MFE-296) carcinoma cell lines and normal breast epithelial cell (MCF-12A) by SRB assay*.

	5a	5b	5c	5d	5e	5f	5g	CPT	5-F Uracil
HUH7	4.64	8.43	7.37	10.29	11.80	15.46	16.35	0.15	30.66
FOCUS	4.15	11.19	6.06	10.81	10.04	11.41	7.77	1>	7.69
MAHLAVU	7.59	9.86	7.35	10.97	14.82	29.46	7.00	1>	9.97
HEPG2	9.37	7.32	7.22	13.00	13.72	18.93	14.61	0.00	5.07
HEP3B	2.49	6.82	1.67	5.59	2.59	8.58	7.03	3.61	15.22
MCF7	9.12	9.05	6.09	8.07	8.47	13.83	10.07	1>	3.51
BT20	18.82	16.92	11.62	42.71	33.94	120.52	18.91	0.07	47.30
T47D	1.91	6.36	0.44	6.45	0.31	8.78	0.85	1>	8.91
CAMA-1	1.48	9.78	1.22	8.07	4.99	13.62	2.73	0.07	1.28
HCT116	10.23	11.49	6.18	12.55	10.89	16.33	8.68	1>	18.67
KATO-3	11.11	10.64	10.07	17.99	17.77	149.55	13.84	1>	ND
MFE-296	24.97	16.51	9.73	34.71	24.59	321.84	18.72	1>	30.68
MCF-12A	5.12	8.06	6.6	23.26	13.91	299.66	12.3	1>	ND

* All the experiments were conducted in triplicate (1 < *R*^2^ < 0.8). ND: not determined.
